# The breathless niche: unrevealing the metabolic landscape of root stem cells under hypoxia stress

**DOI:** 10.1093/plphys/kiad418

**Published:** 2023-08-07

**Authors:** Sebastián R Moreno

**Affiliations:** Assistant Features Editor, Plant Physiology, American Society of Plant Biologists, USA; Sainsbury Laboratory, University of Cambridge, Cambridge CB2 1LR, UK

Stem cells are defined by their ability to self-renew and maintain multipotency. The microenvironment where these cells are located provides the right conditions to maintain the undifferentiated and self-renewable state (Jones and Wagers 2008). Notably, numerous features of stem cell niches are conserved across diverse organisms and tissues.

In animal and plants, low oxygen tension (hypoxia) has shown to be critical to maintain the pluripotent state in stem cell niches (Mohyeldin et al. 2010; Weits et al. 2019). Notably, stem cells not only cope with low oxygen conditions but also benefit from it, probably due to a lower accumulation of DNA mutations (Busuttil et al. 2003) and the prevention of differentiation processes (Ezashi et al. 2005). Although there are some insights into signaling cascades in response to hypoxia in animal cells, the role of hypoxia in plant stem cell niches is still largely unknown.

Interestingly, recent studies have shown how hypoxia affects meristem function by inhibiting the degradation of key regulatory proteins (Weits et al. 2019; Shukla et al. 2019). Although the stem cells are highly hypoxic under normal conditions, adding extra hypoxic stresses to the root causes the disruption of root growth. It was recently shown that the hypoxic state in the quiescent center (QC) in the root apical meristem is controlled by the levels of phytoglobin (heme-containing proteins that oxidize nitric oxide to nitrate). Phytoglobins (*Pgb*) modulate QC maintenance, auxin distribution, and redox state in plants (Mira et al. 2020). Despite these new insights, further analysis is needed into how these plant stem cells respond to hypoxic stress.

In this issue of *Plant Physiology*, Mira et al. (2023) used multi-omic approaches to characterize the hypoxic stress response in the QC stem cells located within the root apical meristem. The authors showed that although QCs are located in an environment with low oxygen levels, additional hypoxic stress triggers degradation of the meristem and root abortion. Notably, the phytoglobin-encoding gene *ZmPgb1.1* diminishes the hypoxic stress in the QC, maintaining a moderate carbohydrate turnover (Fig.). The authors examined cells from hypoxic or normoxic maize root tips using microscopic imaging and enzymatic methods. The results showed that hypoxic stress reduces starch and soluble sugars in the QC at 12 h during hypoxia stress. At 24 h of hypoxia, the authors observed an accumulation of large vacuoles in the proximal initial cells and the disruption of root growth. Notably, overexpression of *ZmPgb1.1* maintained prestressed levels of carbohydrates and small vacuoles even after 24 h of hypoxia.

**Figure.**
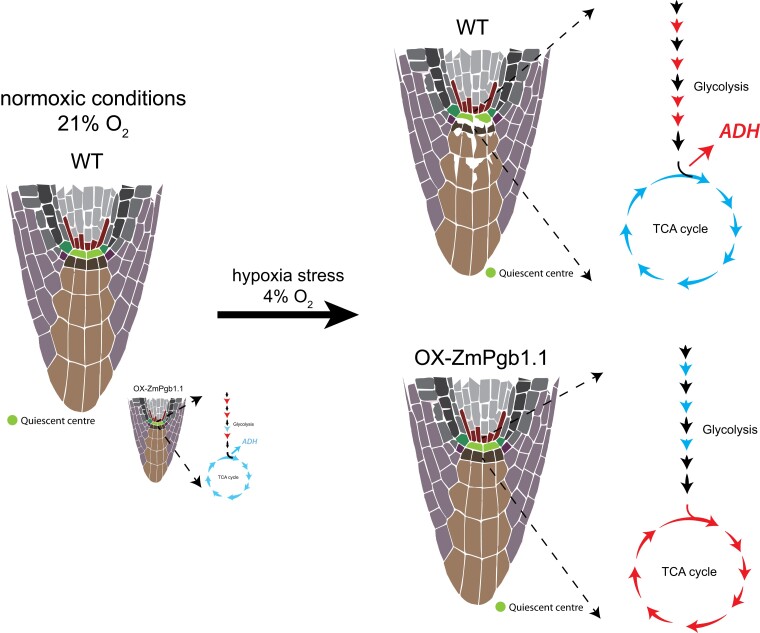
Overview of the phenotypic and metabolic landscape of QC in response to hypoxia stress in WT and ZmPgb1.1 overexpressing line. Hypoxic stress triggers QC degradation in WT plants. Transgenic lines overexpressing ZmPgb1.1 preserve the QC structure with an upregulation of TCA cycle. Red arrows indicate upregulation and blue arrows indicate downregulation. ADH is an hypoxia-responsive gene. The abundance of metabolites in WT during hypoxic stress is relative to normoxic conditions, while the abundance of metabolites in the overexpressing line during hypoxic stress is relative to WT plants during hypoxia.

To understand the carbohydrate turnover previously described and the role of ZmPgb1.1 in this process, Mira and colleagues conducted a multi-omic approach (transcriptomic, proteomic, and metabolomic analysis) in dissected maize QCs at different timepoints under hypoxic conditions in wild-type (WT), suppressed, and overexpressing lines for *ZmPgb1.1*. Remarkably, about 1,800 transcripts and 250 proteins were differentially regulated in *ZmPgb1.1* overexpressing lines before the stress, suggesting that the disruption of this phytoglobin is sufficient to alter the QC stem cell state in normoxic conditions. Metabolites related to central carbon pathways, such as glucose-6-P, fructose-6-P, and 3-P-glycerate, were lowered in the ZmPgb1.1 overexpressing lines. Additionally, a myriad of TCA metabolites such as citrate and aconitate were downregulated in the transgenic line. Interestingly, despite the decreased metabolic levels of citrate and aconitate in low oxygen conditions, the activity of both aconitase and pyruvate dehydrogenase (PDH) was not altered in transgenic lines under normoxic conditions.

The authors observed a very rapid metabolic shift within 12 h of stress in WT plants. Low oxygen altered sucrose/starch metabolism in WT QCs, leading to the accumulation of fructose 1,6-PP, 3-P-glycerate, phosphoenolpyruvate, and lactate and the upregulation of the activity of alcohol dehydrogenase (ADH). Intriguingly, despite the fact that hypoxia increased the activity of ADH, the transcripts and proteins levels were unchanged. This contrasts with what has been observed in intact root tips where low oxygen upregulates *ADH* transcript levels, a classic hypoxia responsive gene. Thus, the authors proposed that ADH is not regulated at the transcriptional level in QCs stem cells in response to hypoxia. Concomitantly, the overall activity of TCA was decreased in response to hypoxia. The enzymatic activity of pyruvate dehydrogenase (PDH), aconitase, and other TCA-related enzymes was drastically decreased in low oxygen conditions. Finally, comparing the different omics profiles between WT, mutant, and overexpressing lines during the hypoxic response, the authors observed that the activity of some TCA-related enzymes such as PDH, aconitase, and 2-OGDH increased in overexpressing lines during the hypoxic response. Overall, the data suggest that *ZmPgb1.1* supresses enzymes associated with fermentation and glycolysis while inducing TCA-related enzymes.

Altogether, the study conducted by Mira et al. (2023) provides a relevant and novel characterization of QCs stem cells in response to hypoxia. The authors showed us clear evidence supporting the idea that despite the fact that stem cells are niched in a hypoxic environment, they are still sensitive to low oxygen stress characterized by a unique metabolic landscape. ZmPgb1.1 was shown to maintain a normoxic state in the QC by maintaining a moderated carbohydrate turnover, probably through the tuning of the TCA cycle. The finding that *ADH*, a classic hypoxia-responsive gene, is not regulated at the transcriptional level in QC emphasizes the distinctive metabolic landscape that stem cell niches may possess compared with other cell types, highlighting the relevance of more omics profiles from stem cell niches. Therefore, this study offers an unprecedented characterization of the metabolic response of root stem cell niches during hypoxia.
